# Barriers and enablers for iron folic acid (IFA) supplementation in pregnant women

**DOI:** 10.1111/mcn.12532

**Published:** 2017-12-22

**Authors:** Kendra Siekmans, Marion Roche, Jacqueline K. Kung'u, Rachelle E. Desrochers, Luz Maria De‐Regil

**Affiliations:** ^1^ HealthBridge Ottawa Ontario Canada; ^2^ Nutrition International Ottawa Ontario Canada; ^3^ Nutrition International Nairobi Kenya

**Keywords:** anaemia, folic acid, iron supplements, prenatal nutrition, primary health care, qualitative methods

## Abstract

In order to inform large scale supplementation programme design, we review and summarize the barriers and enablers for improved coverage and utilization of iron and folic acid (IFA) supplements by pregnant women in 7 countries in Africa and Asia. Mixed methods were used to analyse IFA supplementation programmes in Afghanistan, Bangladesh, Indonesia, Ethiopia, Kenya, Nigeria, and Senegal based on formative research conducted in 2012–2013. Qualitative data from focus‐group discussions and interviews with women and service providers were used for content analysis to elicit common themes on barriers and enablers at internal, external, and relational levels. Anaemia symptoms in pregnancy are well known among women and health care providers in all countries, yet many women do not feel personally at risk. Broad awareness and increased coverage of facility‐based antenatal care (ANC) make it an efficient delivery channel for IFA; however, first trimester access to IFA is hindered by beliefs about when to first attend ANC and preferences for disclosing pregnancy status. Variable access and poor quality ANC services, including insufficient IFA supplies and inadequate counselling to encourage consumption, are barriers to both coverage and adherence. Community‐based delivery of IFA and referral to ANC provides earlier and more frequent access and opportunities for follow‐up. Improving ANC access and quality is needed to facilitate IFA supplementation during pregnancy. Community‐based delivery and counselling can address problems of timely and continuous access to supplements. Renewed investment in training for service providers and effective behaviour change designs are urgently needed to achieve the desired impact.

1

**Key messages**

- The symptoms of anaemia during pregnancy are well known, but many women do not feel personally at risk.
- ANC is the primary delivery channel for prenatal IFA supplements in most countries, but accessibility and delayed first visit remain critical barriers to increased coverage and adherence.
- Health workers require training on prenatal supplementation guidelines and counselling skills to manage side effects and monitor adherence.
- Governments should consider community‐based delivery to improve frequency of contacts and personalized support for women throughout pregnancy.
- Investment in strategic communication is needed to convince women, their families, health workers, and policy makers of the importance and benefits of supplementation for all pregnant women.

## 2 INTRODUCTION

Adequate nutrition during pregnancy is important for improving maternal and child outcomes. Universal iron and folic acid (IFA) supplementation for anaemia prevention among pregnant women is one of the top‐ranked interventions recommended to improve maternal survival (Campbell, Graham, & Lancet Maternal Survival Series Steering Group, 2006). Recent evidence suggests that it may also be associated with improved neonatal and child survival (Nisar, Dibley, Mebrahtu, Paudyal, & Devkota, 2015). In 2012, the World Health Organization (WHO) updated its guidelines for IFA supplementation in pregnant women, continuing to recommend daily oral IFA supplementation (with a dose of 30–60 mg elemental iron and 400 μg folic acid) throughout pregnancy to reduce the risk of low birthweight, maternal anaemia, and iron deficiency (WHO, 2012a, 2015).

Although the efficacy of daily IFA supplementation has been demonstrated, particularly for reducing anaemia (Haider et al., 2013; Pena‐Rosas, De‐Regil, Garcia‐Casal, & Dowswell, 2015), national IFA supplementation programmes in many countries have had difficulty in achieving the high levels of coverage and adherence necessary to effectively reduce anaemia (Sanghvi, Harvey, & Wainwright, 2010; Yip, 1996). In most countries, antenatal care (ANC) services act as the key entry point for delivery of IFA supplements to pregnant women; women who attend ANC are more likely to use IFA supplements, and the number of tablets consumed increases with the number of ANC visits (Klemm et al., 2011; Sununtnasuk, D'Agostino, & Fiedler, 2015). Yet the often poor quality of ANC and lack of attention to nutrition, including inadequate counselling and supplement supply, make it difficult for women to consume the recommended number of IFA tablets during pregnancy (Alam et al., 2015; Galloway et al., 2002; Lacerte, Pradipasen, Temcharoen, Imamee, & Vorapongsathorn, 2011; Lutsey, Dawe, Villate, Valencia, & Lopez, 2008; Stoltzfus, 2011). Consumption of the recommended dose is also often hampered by personal and social barriers, including management of side effects, low priority given to IFA and anaemia, forgetfulness, and conflicting advice where IFA supplement use during pregnancy is not a social norm (Galloway & McGuire, 1994).

With an interest in revitalizing existing IFA programmes, the Micronutrient Initiative (MI) conducted formative research in seven countries in the Africa and Asia regions with high burdens of maternal anaemia and low birthweight to better understand the context‐specific barriers and enablers to improving IFA supplementation coverage and adherence. This paper synthesizes the findings and extracts implications for large‐scale supplementation programmes.

## 3 PARTICIPANTS AND METHODS

Formative research was conducted with mixed qualitative and quantitative methods in selected districts or areas of Afghanistan, Bangladesh, Ethiopia, Indonesia, Kenya, Nigeria, and Senegal between 2012 and 2013 to better understand IFA supplementation knowledge, attitudes, and practices among pregnant women, health care providers, and social influencers, identifying the barriers and enablers associated with coverage and adherence. Local research teams were recruited to carry out the fieldwork in each country and developed and pretested their own adapted versions of the study tools, with technical guidance and quality assurance provided by MI on overall study objectives, study design, sampling, questionnaire development, and reporting of key indicators and themes. Minimum standards for study implementation were assured across contexts, including qualifications of researchers, training duration and content, pretesting of questionnaires, and approval of local adaptations to study protocols.

The studies used a variety of qualitative methods, including focus‐group discussions and key informant or in‐depth interviews, see Table 1 for a description of the methods and respondents in each country. The study instruments were developed in coordination with regional technical advisors and local government, academic and non‐governmental organization partners. In most cases, the formative research studies were designed to address data gaps and deepen the understanding of programme implementation issues in the specific context. Focus group discussions were held with pregnant women or mothers who already had one child. In some countries, focus‐group discussions were also held with husbands and mothers or mothers‐in‐law who were considered key influencers in household decision‐making processes. In‐depth interviews were conducted with women, key influencers, and health care providers, including community health workers. Purposive sampling was used to represent the diversity of target beneficiaries and health providers.

**Table 1:** Overview of formative research studies by region and data collection method

Country and year	Location of study	Methods and respondents
Afghanistan, 2013 (Sharifi, Mohmand, Bahram, & Omar, 2013)	3 districts (Kishm, Bagram, and Surkhroad districts of Badakhshan, Parwan, and Nangarhar provinces)	FGD: PW (*n* = 9 groups), husbands (*n* = 3 groups), mothers‐in‐law (*n* = 3 groups), fathers‐in‐law (n = 3 groups) IDI: health managers (*n* = 9), health workers (3 doctors, 3 midwives, 3 female nurses, 3 community health supervisors), CHWs (*n* = 12), PW (*n* = 18), husbands (*n* = 6), mother‐in‐law (n = 6), father‐in‐law (n = 6), member of Shura Sehi (n = 6)
Bangladesh, 2012 (RTM International, 2012a, 2012b)	4 subdistricts (Kalaroa & Shyamnagar Upazila, Satkhira district; Raipura & Shibpur Upazila, Narsingdi district)	FGD: PW and PPW (*n* = 12 groups), key influential persons (*n* = 4 groups) IDI: PW and PPW (*n* = 20), key influential persons (*n* = 12), health service providers (*n* = 28), health supervisors (*n* = 8), local leaders (*n* = 12)
Indonesia, 2012 (Sartika, 2012)	Lebak and Purwakarta districts	FGD: PW or PPW (*n* = 6 groups), influential persons (*n* = 6 groups) IDI: PW or PPW (*n* = 24), key influencers (*n* = 24), village health workers—midwives or nurses (*n* = 6), facility health workers (*n* = 12), TBA (*n* = 8), cadres or CHWs (*n* = 8), community leaders (*n* = 12), district and provincial level (*n* = 18)
Ethiopia, 2012 (EHNRI & MI Ethiopia, 2012)	8 woredas in Tigray, Amhara, Oromiya and SNNP Regions	FGD: PW attending ANC (*n* = 16 groups), PW not attending ANC (*n* = 16 groups), influential community members (*n* = 8 groups) IDI: health coordinator or supervisors (*n* = 16), HEWs (*n* = 16); MCH clinic nurses (*n* = 8), VCHPs (*n* = 16)
Kenya, 2013 (Center for Behavior Change Communication & Micronutrient Initiative, 2013)	2 districts in Eastern Region	FGD: mothers (3 groups per district), fathers and grandmothers (1 group per district), CHWs (1 group per district) IDI: health workers at selected health facilities (4 per district), national health staff
Nigeria, 2013 (Adegoke & Sambo, 2013)	5 northern states (Jigawa, Katsina, Yobe, Zamfara, and Benue State)	FGD: PPW (gave birth to a child in past year), PW attending ANC, PW not attending ANC, MNCH coordinators, health care providers, influential community members and opinion leaders (*n* = 23) HFS: health workers (*n* = 139 from 93 facilities); health managers (*n* = 29 LGA and 4 managers)
Senegal, 2013 (Faye & Niang, 2013)	2 regions (Dakar, Fatick)	FGD: PW and WRA (*n* = 35 groups) IDI: PW (*n* = 83); WRA or mothers‐in‐law (*n* = 39); husbands (*n* = 27); community volunteers (*n* = 52); health care providers (*n* = 56 from 36 health facilities); managers (*n* = 8)

*Note*. ANC = antenatal care; CHW = community health worker; FGD = focus group discussion; HEW = health extension worker; HFS = health facility survey; HWS = health worker survey; IDI = in‐depth interview; LGA = local government area; MCH = maternal child health; MNCH = maternal, newborn and child health; PPW = post‐partum women; PW = pregnant women; TBA = traditional birth attendant; WRA = women of reproductive age.

Ethical approval was sought by each local partner agency. Informed consent (verbal or written) was obtained from all participants as per recommendation and acceptable standards of each local review board. Voluntary participation and confidentiality were ensured in each of the studies. No remuneration was given. Management of and access to data files followed guidelines from local ethics review boards in each case.

### 3.1 Data analysis

Key features of each country's IFA programme were summarized using the WHO/Centers for Disease Control (CDC) logic model for micronutrient interventions in public health (WHO/CDC, 2011). Where necessary, additional document review (e.g., national policy) and consultation with country representatives helped to fill information gaps.

**Figure 1:**
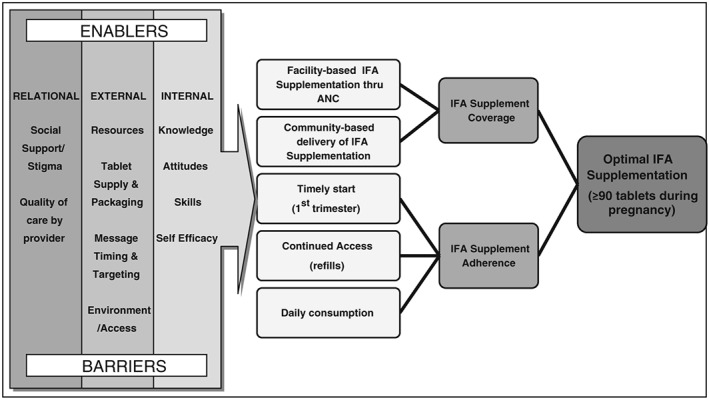
Theoretical framework for IFA supplementation programme impact pathway. IFA = iron folic acid; ANC = antenatal care

The review and synthesis of the formative research results were guided by a socioecological framework blended with an adapted version of the Theory of Triadic influence (Flay, Snyder, & Petraitis, 2009) to identify internal, external, and relational barriers and enablers that impact pregnant women and health providers with regard to target behaviours associated with improved IFA coverage and adherence in the programme impact pathway (see Figure 1). Increasing coverage (defined as receiving any amount of IFA during pregnancy) and adherence (defined as regularly consuming IFA throughout pregnancy as recommended by provider) were considered as two essential outcomes that would contribute to optimal IFA supplementation. Analysis of barriers and enablers to increased coverage focused on the target behaviour of accessing any IFA during pregnancy either through attending ANC services or through community‐based delivery. Target behaviours considered for barriers and enablers to increased adherence included (a) timely access to IFA supplements (starting in first trimester); (b) continued access to IFA supplements throughout pregnancy, requiring regular refills of IFA supplements either through repeat ANC visits or other sources; and (c) daily consumption of IFA supplements.

## 4 RESULTS

### 4.1 Overview of country IFA supplementation programme characteristics

The key features of each country's IFA supplementation programme based on the document review are summarized in Table 2, including the most recent national estimates of ANC and IFA supplementation coverage. Although ANC coverage for at least one visit was very high (>90%) in Indonesia, Kenya, and Senegal, it was quite low (≤55%) in Afghanistan, Bangladesh, and Ethiopia. Over half of pregnant women received any IFA supplementation in Indonesia, Kenya, Nigeria, and Senegal, but much fewer reported receiving more than 90 tablets, a prerequisite for adhering to the recommended consumption.

**Table 2:** Description of IFA supplementation programmes by country

Category or country	Afghanistan	Bangladesh	Indonesia	Ethiopia	Kenya	Nigeria	Senegal
ANC coverage (4+ visits)^a,b^	48% (16%)	55% (26%)	96% (88%)	34% (19%)	96% (58%)	61% (51%)	93% (50%)
IFA coverage (90+ tablets)^a,b^	9%	N/A	76% (33%)	17% (0%)	69% (3%)	63% (21%)	94% (63%)
Policies							
Existing national policies and strategies, including programme guidance	National Reproductive Health Strategy (2012–2016); both IFA & MMNS included in ANC at all types of health facilities in Basic Package of Health Services (2010) Dose regimen: 60 mg Fe + 400 μg FA in last 2 trimesters of pregnancy and first 3 months post‐partum	National Strategy for Prevention and Control of Anaemia (2007); National Guidelines for Prevention and Treatment of IDA (2001) ‐ recommends IFAS for PW Dose regimen: 1 tablet daily 60 mg Fe + 400 μg FA from 2nd trimester until 42 days after delivery	National Food & Nutrition Action Plan 2011–2015 seeks to address maternal anaemia; goal of 85% PW receive 90 iron tablets by 2015; Plan of Action on Community Nutrition (2010–2014) includes IFA for PW in “community nutrition pillar” Dose regimen: 60 mg Fe + 250 μg FA	National Nutrition Strategy (2008) target: 50% of mothers get IFAS for >90 days during pregnancy and post‐partum period by 2015. National MN Guidelines (2004) recommend daily IFAS for at least 6 months during pregnancy and 3 months post‐partum. National Nutrition Programme (2013–2015) includes routine IFA/MMN supplementation for PLW	Food & Nutrition Security Policy (2011); National Nutrition Action Plan (2011–2017); National Technical Guidelines for MND Control recommends daily provision of 60 mg Fe + 400 μg FA for PW from first month of pregnancy for 6 months. If dose not achieved during pregnancy, continue during post‐partum period for 6 mo or increase dose to 120 mg Fe in pregnancy.	National Strategic Health Development Plan 2010–15; Integrated MNCH Strategy (2007) calls for focused ANC package, includes prevention & treatment of iron deficiency. Roll‐out of package at state level is variable. MND Control Guidelines recommends IFAS for PW Dose regimen: 30–60 mg Fe + 400 μg FA	Plan National de Développement Sanitaire (2009–2018) includes universal coverage of IFAS for all women who seek ANC from public health centres. Politiques, Normes et Protocoles en Santé de la Reproduction recommends IFAS starting at first ANC visit in first trimester Dose regimen: 60 mg Fe + 400 μg FA
Production and supply							
Adequacy of supply (type of IFAS used in country and function of supply chain system)	IFA & MMN tablets included as essential drugs for BPHS. IFAS supply at health facilities often does not match with recommended doses. Most health facilities in project areas receive essential drug supplies on a quarterly basis from provincial office of the NGO implementing the BPHS. Main problem is insufficient quantity of medicines (supply not based on need).	Uncoated IFA tablets are packaged in paper. Supply chain varies by distribution channel: (a) National Nutrition Programme: demand for IFA comes from NGOs and supplied through Central Medical Stores, (b) DGHS: procure IFA through Sr. Store Officer based on amount procured the previous year, and (c) DGFP: Drug & Dietary Supply kits contain 2000 IFA tablets each, obtained from Essential Drugs Company	Uncoated IFA tablets produced by Kimia Farma at national level; packaged into 30 tabs per sachet at district level. Supply chain varies by province: (a) Central distribution annually through District Health Office to health centres, quantity based on number of PW and (b) province receives annual budget allocation for purchase of IFA tabs through bidding process, IFA distributed monthly or quarterly from district to health centres.	Zonal Health Dept. supplies the Woreda Health Office that provides IFA to cluster health centres that distribute to health posts. In project areas, type of IFA tablet varies by health facility and region; majority use ferrous sulphate with folic acid.	IFA tablets procured by Kenya Medical Supplies Agency and distributed via essential drug kit to public health facilities through regional and district depots (“pull” system for higher level facilities, “push” system for health centres and dispensaries). MOH recently introduced enteric coated combined iron (60 mg) and folic acid (400 μg) tablets.	State Central Medical stores procure and distribute to LGA; LGA drug store distributes to all functioning health facilities providing ANC services within the Ward. Revised draft of National Essential Medicine List specifies enteric coated combined IFAS formulation but only used by 1 of 5 states assessed.	Supply purchased centrally (Pharmacie Nationale d'Approvisionnement) and sent to regional depots for distribution to periphery health facilities, weak inventory management and lack of funds to place orders at the peripheral level results in irregular supply of IFA tablets. Parallel supply channels of other development partners exist, lack of coordination results in competition and duplication of effort.
Delivery							
Primary and secondary delivery systems in project area at time of formative research (any restriction on who can deliver IFA, any involvement by community‐based personnel)	IFAS provided by midwives, nurses and doctors at health facilities during ANC visits.	IFAS provided as part of ANC services through Satellite Clinics, Maternal & Child Welfare Centres, and Community Clinics.^c^ Several national & international NGOs and private sector also distributing IFA to PLW	Nationally, IFAS provided through ANC by nurses, midwives, and village midwives. In project areas, most IFAS delivered by village midwives at ANC visits in Posyandu; CHWs and TBAs help distribute IFA to PW at community level.	IFAS provided through ANC visits at health facilities by midwives, HEWs or nurses. Home‐based ANC and outreach ANC‐PMTCT approaches piloted in Amhara and Oromiya regions, respectively.	IFAS routinely delivered as part of focused ANC at MCH clinics, also distributed in semiannual Child Health Week campaigns. CHWs were not allowed to dispense drugs, policy under review.	IFAS delivered during ANC at health facilities; revised guidelines allow community‐based delivery by CHWs, and this is being piloted in northern states.	Primary delivery through ANC at health facilities; women were given prescriptions to purchase IFA tablets from pharmacists. Midwives are most frequent prescribers (85%). No official delivery by CHWs, piloted by PRN project.
Strategy for management, training (frequency & quality), and maintaining motivation among providers and distributors	Health facilities staff and CHWs have not received specific training on counselling or communication of IFAS; no IFAS‐specific job aids (guides, manuals).	Topic of anaemia as part of nutrition is addressed in training of FWVs (18 months of basic training), FWAs (1 month) and SBAs (6 month training programme).	Village midwives trained on general MCH but not IFAS specifically. Refresher training provided for half or 1 day on regular basis. IFAS part of MCH services package; no additional incentive given for this activity.	Rely on preservice training with no refresher sessions, expect supportive supervision from HEW supervisors. Across study woredas, many HEWs, MCH clinic nurses and HEWs supervisors reported no additional training on ANC or IFAS.	Different cadres of health workers receive preservice and in‐service training on IFAS. Limited pre and in‐service training on focused ANC, contributing to low quality of counselling by care providers.	Health workers received no previous training on IFAS delivery mechanism or counselling; some received training on BCC for IFAS.	No data on training provided to health care providers. Inconsistent and infrequent supervision of health care providers.
Quality							
Existence of any external or internal quality control system	No data found	Lack of central coordination and oversight; no mention of any quality control systems in place	Midwives coordinator conducts “impromptu inspections” to assess village midwife performance, including IFA consumption among PW	None specific to IFAS, not perceived as important aspect of ANC to be monitored for quality control	Checklist for IFAS developed (2013) for use by health managers during scheduled supportive supervision visits to health facilities	Periodic monitoring, supportive supervision of LGA health workers by senior health workers, and managers from the states	Lack of quality control system; National Lab for Quality Control of Drugs does not check IFA tablets available on the market, also need for enforcing product price controls at both public and private levels.
Behaviour change communication							
Intervention strategy for behaviour change (include message delivery by community‐based personnel here)	PW receive ANC card to monitor progress, IFAS and next visit date. Health facilities conduct group education sessions, but IFAS is not well covered; individual anaemia counselling done on case by case basis. CHWs do home visits for education based on client needs.	Posters and other materials developed by the government, donor agencies and NGOs. The media remains underutilized for promotion of activities to tackle anaemia. (Rashid, Flora, Moni, Akhter, & Mahmud, 2010)	MCH booklet for PW explains anaemia & IFAS; no other IFAS BCC materials or budget. Village midwives track adherence, ask CHWs, family members to remind PW to take IFA. Midwife‐led PW groups provided context to discuss MCH matters but discontinued for budget reasons.	Village community health promoters actively identify and refer PW to health posts for ANC services.	No national or coordinated BCC strategy at time of this study, but National IFAS Communication Strategy (2013–17) launched in 2013. CHWs play role in social mobilization. Poor monitoring and follow up of clients due to inadequate info in client‐held records (ANC cards).	Societal Mobilization, Advocacy and Communication strategy developed and aligned with National Nutrition Plan. Semiannual MNCH Week instituted in 2010 to promote key health messages.	Midwives or CHWs lead group education talks on variety of reproductive health topics (including IFAS) before ANC sessions at health facility level; CHWs also conduct group info sessions and home visits of PW in communities where they are active.
Monitoring and evaluation							
Data on coverage and adherence (HMIS indicators, national surveys, or evaluation of IFAS programme coverage or effectiveness)	National Nutrition Survey (2013) and MICS (2010–11) include ANC coverage and anaemia in PW	HMIS include ANC coverage and anaemia as 1 of 10 reported diseases; no data on IFA coverage or adherence. DHS does not track number of IFA tablets received by women.	Provinces use M&E funds for nutrition (including IFAS) to monitor IFA stock and coverage but not adherence. DHS (2012) includes ANC/ IFA coverage & adherence	HMIS monitors ANC coverage and frequency but no specific indicator of ANC services, including IFAS. Minimal attention given to ANC content and service quality. DHS (2011) includes ANC/ IFA coverage and adherence	District HMIS tracks 11 core indicators on MN (including anaemia cases, ANC visits, and IFAS). DHS, Service Provision Assessment, and National MN Survey include ANC/IFA coverage and adherence	HMIS monitors ANC coverage and frequency, IFA coverage, and adherence. Few facilities provide timely or accurate data on IFAS in study area. DHS and MICS include ANC or IFA coverage and adherence	IFAS monitoring included as part of ANC provision; focus on prescription given but no indicator of adherence. DHS includes ANC or IFA coverage and adherence.

*Note*. ANC = antenatal care; BCC = Behaviour Change Communication; BPHS = Basic Package of Health Services; CHW = community health worker; Dept = department; DGFP = Directorate General of Family Planning; DGHS = Directorate General of Health Services; FA = folic acid; FWA = family welfare assistant; FWV = family welfare visitor; Govt = government; HEW = health extension worker; HMIS = Health Management Information System; IFA = iron folic acid; IFAS = iron folic acid supplementation; LGA = local government areas; M&E = monitoring and evaluation; MCH = maternal child health; MICS = Multiple Indicator Cluster Survey; MMN = Multiple Micronutrient; MNCH = maternal, newborn and child health; MND = micronutrient deficiency; MOH = Ministry of Health; NGO = nongovernmental organization; PLW = pregnant and lactating women; PMTCT = Prevention of Mother‐to‐Child Transmission of HIV; PW = pregnant women; SBA = skilled birth attendant; TBA = traditional birth attendant.

ANC coverage defined as receiving antenatal care at least once from a skilled provider; IFA coverage defined as taking any iron tablets during the pregnancy of their last birth.

Source: Afghanistan National Nutrition Survey 2013 (Ministry of Health (Afghanistan) & Unicef, 2013); Bangladesh DHS 2011 (National Institute of Population Research and Training (NIPORT), Mitra and Associates,, & ICF International, 2013); Indonesia DHS 2012 (Statistics Indonesia (Badan Pusat Statistik—BPS), National Population and Family Planning Board (BKKBN), Kementerian Kesehatan (Kemenkes—MOH),, & ICF International, 2013); Ethiopia DHS 2011 (Central Statistical Agency [Ethiopia] and ICF International, 2012); Kenya DHS 2014 (for ANC coverage) (Kenya National Bureau of Statistics, Ministry of Health, National AIDS Control Council, Kenya Medical Research Institute,, & National Council for Population and Development, 2015) & 2008 (for IFA coverage) (Kenya National Bureau of Statistics (KNBS) & ICF Macro, 2010); Nigeria DHS 2013 (National Population Commission (NPC) [Nigeria] & ICF International, 2014); Senegal DHS 2010 (Agence Nationale de la Statistique et de la Démographie (ANSD) [Sénégal] & ICF International, 2012a).

Personnel involved (a) Family Welfare Visitors at Satellite Clinics and Maternal and Child Welfare Centres; (b) female welfare assistants, health assistants and Community Health Care Providers (CHCP) at Community Clinics.

National policies for IFA supplementation are in place for all countries, with most recommending a daily dose of 60 mg elemental iron and 400 μg folic acid for at least 90 days during pregnancy. In Afghanistan, Bangladesh, and Ethiopia, IFA supplementation is recommended to continue into the post‐partum period. Most countries procure IFA supplements at the national level and distribute them to regional and district levels. Issues with sufficient quantities being forecast and distributed result in periodic stock‐outs at primary health facilities. Although the enteric coated combined IFA tablet (WHO, 2012a) was recently introduced in Kenya and Nigeria, the type of supplement procured by other countries varies. Facility‐based ANC was the primary delivery platform for IFA supplementation in all the countries included in this study. Afghanistan, Bangladesh, and Indonesia also have established community‐based delivery systems (WHO, 2012b); pilot projects in Ethiopia and Senegal have tested delivery through community health workers.

Outside of routine supervision within the health system, evidence of quality control systems for IFA supplementation was not found. In Kenya and Senegal, the Service Provision Assessment surveys have provided useful data on availability and quality of ANC services, including IFA supplementation (Agence Nationale de la Statistique et de la Démographie (ANSD) [Sénégal] & ICF International, 2012b; National Coordinating Agency for Population and Development (NCAPD) [Kenya], Ministry of Medical Services (MOMS) [Kenya], Ministry of Public Health and Sanitation (MOPHS) [Kenya], Kenya National Bureau of Statistics (KNBS) [Kenya], & ICF Macro, 2011). Monitoring and evaluation of IFA supplementation programmes are done through national health management information systems and periodic demographic and health surveys. However, emphasis tends to be placed on tracking coverage of ANC (not type and quality of services provided) and IFA supplementation (not adherence).

Behaviour change interventions for IFA supplementation during pregnancy were minimal in most countries. ANC cards and MCH booklets provide written information about IFA supplementation, although the effectiveness of this communication method was questioned by service providers in our study. In most countries, CHWs are tasked with social mobilization and behaviour change communication message delivery for pregnant women to encourage ANC attendance and IFA supplement use.

### 4.2 Barriers and enablers to coverage

#### 4.2.1 Women's knowledge about anaemia and taking IFA during pregnancy

Awareness of the signs and symptoms of anaemia during pregnancy was very high among women in every country. Yet knowledge of the causes of anaemia and ways to prevent it were more variable, as was an individual woman's self‐risk perception, without symptoms. Anaemia was often attributed to a poor diet and hard work. Consistent with this dietary aetiology of anaemia, both women and health care providers believed that eating a more nutritious diet would help to prevent and treat anaemia during pregnancy. When asked specifically about the purpose of supplementation, women believed that IFA supplements prevent anaemia (Afghanistan, Kenya, Nigeria, and Senegal), improve normal growth and delivery of the baby (Afghanistan and Senegal), help them stay healthy (Indonesia, Kenya, and Senegal), and reduce dizziness and fatigue (Afghanistan, Indonesia, and Kenya).

#### 4.2.2 Women's perceived barriers or enablers to receiving IFA through ANC

Women were asked about their beliefs, attitudes, and practices with regard to ANC, the primary delivery platform for IFA supplementation in these countries. Although attending ANC at least once during pregnancy was very common, various reasons were given for not attending ANC frequently or only late in pregnancy. Some women interviewed in Ethiopia, Kenya, and Senegal perceived ANC as primarily for curative purposes (dealing with pain, complications, or illness) and did not feel the need to go if they were having a healthy pregnancy. Kenyan women also spoke of fears of attending ANC, such as fear of hospitals, HIV testing, or pregnancy confirmation. Low perceived value and need for ANC visits during pregnancy was an important internal barrier among women in Bangladesh.

The most commonly reported external barrier to IFA supplementation coverage through ANC was accessibility, both geographic accessibility of health facilities providing ANC services and economic factors such as the cost of ANC consultations and IFA supplements. The high cost of ANC consultations was a major barrier for women in Senegal, despite the low cost of IFA tablets relative to the higher cost of other ANC services. Frequent unavailability of the health extension worker demotivated women in Ethiopia to attend ANC. In Bangladesh, lack of supplies at government health facilities and absence of the doctor to prescribe the tablets were cited as barriers. In contrast, women in Afghanistan perceived government health facilities as accessible overall and cited various advantages to ANC provided there, including the availability of female doctors and midwives, free consultation services and medicines, and attendance by their peers. However, long waiting times and the cultural necessity of being accompanied by a family member for ANC consultations were barriers in this context.

In terms of relational barriers and enablers to attending ANC, there was general agreement that historical social barriers to ANC attendance were no longer an issue. Yet women were still highly influenced by family members. In Afghanistan, Nigeria, and Senegal, the decision to attend ANC was made by other family members, usually the husband and/or mother‐in‐law, not women themselves. In Bangladesh and Nigeria, some older adult women discouraged younger women from attending and using medicines during pregnancy. Kenyan women described their husbands as being generally supportive but a minority felt that these men did not value ANC or discouraged them from early ANC attendance. Perceived low quality of ANC service delivery and poor treatment by health care providers were also important relational barriers. In Bangladesh, women did not receive adequate counselling or understand the messages given by health workers. In Ethiopia, women saw ANC as synonymous with injections and felt that limited services were provided. Senegalese respondents felt the offered ANC services did not meet expectations, but some women were still motivated to go in order to establish a relationship with the midwife as a means of accessing support during labour and getting a birth certificate afterwards.

#### 4.2.3 Women's perceived barriers or enablers to receiving IFA through community delivery platforms

Community‐based delivery of IFA supplementation was rare in the areas included in this study. In Indonesia, village midwives and community health volunteers were an important delivery channel for IFA tablets for pregnant women. In Afghanistan, where some community‐based delivery was reported, women had concerns whether community health workers and traditional birth attendants had received sufficient training. They felt that community‐based providers were “illiterate, not knowledgeable, not a doctor”. Yet the proximity of these service providers was seen as a major enabler to improve access and thus coverage of IFA.

#### 4.2.4 Health workers' perceived barriers or enablers to provide IFA supplementation during ANC

Lack of IFA supplementation‐specific training and capacity gaps were reported by health care providers in all countries, summarized in Table 3. In Nigeria, health personnel were either not aware of existing guidelines or had no access to them, relying on their own experience to make care decisions as opposed to using national evidence‐based guidelines. In Senegal, midwives (the most frequent prescribers of IFA supplements to pregnant women) had a high level of knowledge regarding anaemia and IFA supplementation but varied in their prescription practices due to perceived lack of value to the mother in early pregnancy and concerns with nausea during the first trimester. Reliance by health workers on signs and symptoms of anaemia for diagnosis as well as lack of awareness regarding IFA being recommended for all pregnant women was also identified in interviews.

**Table 3:** Gaps identified by health care providers through formative research

Country	Training needs
Afghanistan	• Identified need to improve counselling offered through ANC and home visits • Need training in stock monitoring and supply chain management • Inadequate knowledge on IFA, weak distribution, monitoring, and counselling
Bangladesh	• Need to improve capacity of service providers to administer IFA, monitor supply and track individuals on supply and utilization, provide quality counselling • Need to strengthen capacity of supply chain managers in forecasting
Indonesia	• Village midwives trained on general MCH topics but not IFA supplementation specifically • Need to improve capacity of health centre staff, village midwives, and CHWs to counsel pregnant women in interpersonal communication and group counselling • Need for regular supportive supervision
Ethiopia	• No in‐service or refresher training • Insufficient supportive supervision from HEW supervisors to help build capacity • Lack of knowledge and interest in IFA programme among health workers
Kenya	• “Limited” level of preservice and in‐service training on focused ANC and IFA supplementation (per Kenya Services Provision Assessment) contributing to low quality of counselling by health workers • Incomplete District Health Information System reporting by facilities and inadequate data analysis, feedback, and reviews by concerned focal persons at district, regional, and national levels • Capacity building on IFA service delivery needed for both facility‐based and community‐based health workers (coverage a major challenge identified)
Nigeria	• No specific IFA supplementation training provided to health workers • Need training in counselling on management of side effects and encouragement of adherence, using new guidance provided by government • Need capacity strengthening in point of delivery supply management and forecasting
Senegal	• Need for training CHWs to distribute IFA, especially counselling skills to improve adherence • Need for training health workers to train, support, and monitor CHWs in IFA distribution

*Note*. ANC = antenatal care; IFA = iron folic acid; MCH = maternal child health; CHW = community health worker; HEW = health extension worker.

Inadequate supply of IFA supplements was cited as a barrier by health care providers in every country. In most cases, the supply issue was seen as a problem of inadequate procedures for procurement at the local level instead of inadequate stocks at upper or central levels. In Senegal, respondents described concerns that women were purchasing poor quality IFA supplements from other sources due to variability in the price of supplements and frequent stock outs at health facilities.

In Ethiopia, health care providers reported growing demand for IFA supplements by women attending ANC and through community‐based services as enabling higher IFA coverage. Health extension programme strengthening, the versatile role of Voluntary Community Health Promoters in social mobilization, community education and identification of pregnant women for ANC, as well as the strong relationships between health extension workers and pregnant women were also cited as enabling factors.

### 4.3 Barriers and enablers to adherence

#### 4.3.1 Women's perceived barriers/enablers to timely access to IFA supplementation

Barriers to timely initiation of IFA supplementation were closely related to those for seeking ANC in the first trimester. Few women knew the importance of or saw value in seeking ANC during the first trimester. Most women thought the first ANC visit was recommended around the third month of pregnancy (beginning of 2nd trimester), but some felt that ANC was only needed if a problem came up. Women in Ethiopia and Senegal expressed reluctance to reveal the pregnancy publicly during the first trimester; women in rural areas of Senegal in particular believed that this would put the fetus at risk (“some pregnancies cannot support being known”). Relational barriers to early ANC attendance included various social implications of revealing a pregnancy and requiring the support of their husband and/or mother‐in‐law. Health care provider reluctance to prescribe IFA supplements during the first trimester was also observed in several contexts.

Women's perceptions of the purpose and value of IFA supplementation also influenced when they believed it was important to start taking the tablets. In Senegal, women perceived iron supplements as useful for restoring blood, avoiding complications during delivery, and giving strength for the delivery. Under this paradigm, it made sense for pregnant women to take the supplements later in the pregnancy in preparation for the delivery. The utility of IFA supplementation for anaemia prevention or treatment of asymptomatic anaemia was less well understood or important to these women.

#### 4.3.2 Women's perceived barriers/enablers to receive regular refills of IFA supplementation

Accessing at least 90 tablets, and preferably 180 tablets, during the course of the pregnancy requires multiple ANC visits or accessing IFA tablets from other sources, including community‐based providers. In general, women did not know the recommended number of ANC visits or duration of IFA supplementation, relying on the instructions given by health care providers as to when to return for the next ANC visit. Although multiple ANC visits were perceived as the norm by respondents in Kenya, Nigeria and Senegal, actual practices varied widely across countries. External barriers to continued access to IFA supplements included financial constraints related to travel to ANC clinics for women in Kenya, receiving inadequate number of IFA tablets from health facilities in Ethiopia and stock‐outs at health facilities in Senegal that required filling prescriptions at pharmacies at an additional cost and inconvenience, decreasing the likelihood that she will get them. Relational factors also played a role, such as in Senegal, where many women relied on their husbands to provide money to attend ANC and purchase supplements.

#### 4.3.3 Women's perceived barriers or enablers to consume recommended number of tablets

The most common internal barriers to women consuming the recommended number of tablets during pregnancy were misconceptions about IFA supplementation and its benefits during pregnancy, experience of side effects, and forgetfulness. Various misconceptions about IFA supplements and anaemia hindered women's willingness to take them. A woman in Ethiopia said “It is only given for ill women. I don't know why it is given to us.” Nigerian women reported that some women believed that taking IFA can pose a danger to the mother and child and makes babies big and causes difficulties for women during labour. However, knowing the benefits of IFA supplements enabled others to continue taking them, as one woman from Nigeria stated “I don't forget [to take IFA supplements] because I understand its importance … those who do not understand the importance will stop.” Experiencing negative side effects of IFA supplementation was reported to varying extents by women as a barrier to regular consumption of IFA tablets, yet many reported self‐efficacy in managing the side effects, with strategies coming from both health care providers and peers.

External barriers to regular consumption included financial accessibility and insufficient supply of tablets, as described in the previous section. Access to free IFA supplements enhanced adherence in Nigeria. In Indonesia, the generic IFA tablets provided at health facilities had unappealing attributes, in addition to side effects. Village midwives in that context encouraged women to purchase brand name tablets to improve adherence. In Senegal, women reported specific preferences for the syrup or tablet form of supplements and attributed the lack of their preferred form as a barrier to continued consumption.

Relational factors were reported to improve adherence. This included family and community support in Nigeria; reminders from family members to take supplements daily helped to reduce forgetfulness. In Senegal, positive relationships between pregnant women and midwives resulted in women returning to the health facility for advice when they experienced side effects.

#### 4.3.4 Health workers' perceived barriers or enablers to providing recommended number of IFA tablets

A major barrier to pregnant women regularly consuming IFA tablets is the inconsistent prescription practices of health care providers. When asked about IFA supplement dosage and duration, health workers responses varied both within and across countries. Furthermore, health workers seemed to have inadequate tools and skills in counselling to support and monitor adherence. In Senegal, despite adequate knowledge of anaemia and IFA supplementation, 40% of prescribers did not give advice to women when they prescribed IFA supplements. In Bangladesh, community health workers reported that women were only given 20 tablets each time, and refills were hampered by inadequate stocks. Lack of promotional materials and job aids at health facilities were also cited as barriers to improving adherence.

## 5 DISCUSSION

In synthesizing the formative research on prenatal IFA supplementation programmes in selected districts of seven countries across Asia and Africa, we identified both challenges and opportunities for revitalizing existing programmes that have implications whether IFA or multiple micronutrients are to be used. This requires strategies that address both individual‐level beliefs and behaviours as well as social and institutional factors.

Learning how internal or personal factors impact women's experience with ANC and IFA supplements helps to inform programmes. Recognizing and valuing the potential for micronutrient supplements to directly and positively impact their own health, especially in early pregnancy, are important for increasing acceptance and demand for this intervention among pregnant women. These perceived benefits are necessary to outweigh the social, financial, and resource costs and physical discomforts currently associated with IFA supplements. Other studies have shown a positive association between the perceived health benefits of micronutrient supplementation and increased coverage and adherence (Aikawa et al., 2006; Galloway et al., 2002; Klevor et al., 2016; Lutsey et al., 2008; Nechitilo et al., 2016; Zavaleta, Caulfield, Figueroa, & Chen, 2014). However, the apparent difficulty in convincing pregnant women in our study contexts of the therapeutic and preventive value of iron supplements for anaemia also raises important questions regarding what key messages can be used to promote supplementation, especially as countries shift toward recommending multiple micronutrient supplements (Haider & Bhutta, 2015). Greater investment in strategic communication is recommended so that pregnant women and health care providers understand and believe in the importance of supplementation for all pregnant women.

Reducing systemic barriers to accessing the supplements is critical for improving coverage and adherence. Where ANC remains the primary point of distribution of IFA supplements to pregnant women, our findings point to a need for strengthening ANC demand, especially in the first trimester (Klemm et al., 2011). Although increased acceptance of the value of ANC was evident, gaps remain in terms of ensuring all pregnant women access these services in a timely and regular manner. Behaviour change interventions are needed that emphasize the value of preventative ANC services, the value of first trimester attendance, and knowledge of the specific services to be provided during these visits. Innovative approaches to reaching women prior to conception, such as through marriage registries (Jus'at et al., 2000), mass media campaigns, or the private sector are needed.

The need for strengthening the delivery of IFA supplementation through improved quality of maternal health care provision is also evident. Service providers need to have the knowledge and motivation to adequately distribute, inform, and problem solve with the target population (WHO/CDC, 2011). Our results highlight major gaps in provider training within existing IFA supplementation programmes; facility‐ and community‐based health workers were ill‐equipped to provide this intervention. In addition to improving the quality of ANC services overall, specific training is needed on the importance of taking IFA throughout pregnancy and IFA supplementation guidelines, including timing, dosage, frequency, and proactive counselling to manage side effects and actively monitor adherence. Health worker training should focus not only on the content of messages but also on the counselling and interpersonal skills needed to foster positive relationships with pregnant women and their family members (Nechitilo et al., 2016). Enhanced monitoring and evaluation of IFA coverage and adherence, including more accurate measurement in national surveys of actual consumption of IFA tablets by women during pregnancy, are also needed.

Addressing supply chain management issues and improving product stock monitoring are also needed to ensure the availability of supplements for women, especially in contexts where the tablets are provided free of charge. Procurement of high quality tablets is also expected to improve adherence because our study and others have shown that the colour, size, coating, and packaging of iron tablets can affect consumption behaviour (Galloway et al., 2002).

Improving continued access to supplements throughout pregnancy is also a priority as fewer prenatal visits are associated with a lower number of IFA tablets consumed (Lutsey et al., 2008; Onyeneho et al., 2016; Sununtnasuk et al., 2015). In Vietnam, the most important factor enabling taking iron tablets for at least 5–9 months was a frequent supply of iron tablets (Aikawa et al., 2006). Establishing community‐based delivery of IFA supplements and/or community health workers providing follow‐up are key components of the strategies used in Indonesia, Nepal, Nicaragua, and Thailand to successfully increase IFA supplementation coverage (Pokharel, Maharjan, Mathema, & Harvey, 2011; Sanghvi et al., 2010). Community‐based distribution of IFA supplements was positively viewed by the women in our study.

A delay in the first ANC visit has been shown to be associated with lower haemoglobin levels (Lutsey et al., 2008). Due to personal and social ramifications of revealing a pregnancy too early, programmes seeking to encourage early adoption of micronutrient supplementation in pregnancy should explore alternative sources of supplements that are easily accessed at the community level. Distribution of IFA and referral to ANC are performed at the community level by Female Community Health Volunteers in Nepal as part of an integrated delivery platform, where adherence and coverage rates are among the highest (Pokharel et al., 2011). Recent evidence from Nepal shows an association between IFA and newborn or child survival, with the greatest effect in women who started taking IFA early in pregnancy and took 150–240 supplements (Nisar et al., 2015). Yet regardless of the distribution point, supply‐side issues of adequate supplies of quality IFA tablets and distributing them in adequate amounts to women must also be addressed (Ayoya, Bendech, Zagre, & Tchibindat, 2012; Galloway et al., 2002).

Strategies involving multiple stakeholders are required to support improved adherence with IFA supplementation in order to address the various barriers expressed by women in this study. Creating an environment where it is normalized, valued, and prioritized for a pregnant woman to take “one tablet a day” and “take iron supplements throughout pregnancy” will require broad efforts, starting with renewed political commitment and, in some cases, removal of restrictions on who can provide the supplements (WHO, 2012b). Advantages to community based platforms include easier access to refills, personalized counselling on managing side effects and opportunities for educating and involving other key decision makers in the household. The critical role of the family for social support and advice during pregnancy has been observed by others (Martin et al., 2016; Matare, Mbuya, Dickin, Humphrey, & Stoltzfus, 2015; Wulandari & Klinken Whelan, 2011). Engaging influencers in the community, such as husbands and mothers or mothers‐in‐law, in health promotion activities for ANC attendance, and micronutrient supplementation during pregnancy, is expected to address some of the barriers at the household decision‐making level observed in this study. Engaging family members as adherence partners (Martin et al., 2016) or using innovative technology for reminder messages (Matiri, Pied, Velez, Cantor, & Galloway, 2015) can also improve adherence.

## 6 CONCLUSION

In each context studied, specific cultural beliefs and practices were identified that act as barriers to IFA supplement consumption. Formative research is essential prior to the development of behaviour change interventions to design context‐specific interventions that motivate pregnant women, their families, and health care providers to increase access to and consumption of IFA supplements. In addition, to improved facility‐based ANC access and quality, community‐based delivery and counselling have the potential to address concerns found in IFA supplementation programmes, with earlier contact, potential for frequent resupply and more personal support for pregnant women. Renewed investment in prenatal supplementation programmes with strong behaviour change interventions at policy, provider, community, and individual levels is urgently needed to achieve the World Health Assembly nutrition global targets for anaemia and low‐birthweight reduction.

## CONFLICTS OF INTEREST

KS and RD do consultancy work for Nutrition Intermational (formerly the Micronutrient Initiative). MR, JK and LMD are employees of Nutrition International (formerly the Micronutrient Initiative).

## CONTRIBUTIONS

MR and JK conceptualized and designed the study with input from KS. KS and RD analysed the data. KS prepared the manuscript. All authors reviewed and contributed to the final version.
